# Piperine metabolically regulates peritoneal resident macrophages to potentiate their functions against bacterial infection

**DOI:** 10.18632/oncotarget.5957

**Published:** 2015-10-02

**Authors:** Hao Pan, Li-Hui Xu, Mei-Yun Huang, Qing-Bing Zha, Gao-Xiang Zhao, Xiao-Feng Hou, Zi-Jian Shi, Qiu-Ru Lin, Dong-Yun Ouyang, Xian-Hui He

**Affiliations:** ^1^ Department of Immunobiology, College of Life Science and Technology, Jinan University, Guangzhou, China; ^2^ Department of Cell Biology, College of Life Science and Technology, Jinan University, Guangzhou, China; ^3^ Department of Fetal Medicine, the First Affiliated Hospital of Jinan University, Guangzhou, China

**Keywords:** bacterial infection, mTORC1, peritoneal macrophages, piperine, SLC7A5/SLC3A2, Immunology and Microbiology Section, Immune response and Immunity

## Abstract

Pepper, a daily-used seasoning for promoting appetite, is widely used in folk medicine for treating gastrointestinal diseases. Piperine is the major alkaloid in pepper and possesses a wide range of pharmacological activities. However, the mechanism for linking metabolic and medicinal activities of piperine remains unknown. Here we report that piperine robustly boosts mTORC1 activity by recruiting more system L1 amino acid transporter (SLC7A5/SLC3A2) to the cell membrane, thus promoting amino acid metabolism. Piperine-induced increase of mTORC1 activity in resident peritoneal macrophages (pMΦs) is correlated with enhanced production of IL-6 and TNF-α upon LPS stimulation. Such an enhancement of cytokine production could be abrogated by inhibitors of the mTOR signaling pathway, indicating mTOR's action in this process. Moreover, piperine treatment protected resident pMΦs from bacterium-induced apoptosis and disappearance, and increased their bacterial phagocytic ability. Consequently, piperine administration conferred mice resistance against bacterial infection and even sepsis. Our data highlight that piperine has the capacity to metabolically reprogram peritoneal resident macrophages to fortify their innate functions against bacterial infection.

## INTRODUCTION

Pepper has long been used as a food seasoning and an ingredient in folk medicine. Modern medicinal chemistry has revealed that piperine is the major plant alkaloid in pepper. This alkaloid possesses a broad spectrum of pharmacological activities, including anti-depression and anti-epilepsy effects [1, 2], as well as abilities to reduce lipid deposition and body weight [3]. It is also effective for a variety of experimental inflammatory diseases, including gastric ulcer [4], arthritis [5], and endotoxin-induced shock [6]. In Asian countries, pepper is broadly used as a medicinal herb for treating gastrointestinal diseases [7], in which piperine might act as the major active component. Therefore, piperine appears to be a potential immunomodulator for therapeutic usage against inflammatory diseases as well as bacterial infection. However, the relationship between the metabolic and pharmacologic effects of piperine is unclear; particularly, the underlying action mechanism of piperine in the setting of bacterial infection is largely unknown.

Considering that pepper acts as a seasoning in improving digestion and appetite, we have tried to test influences of its major component piperine on a variety of signaling pathways of cellular metabolism. One of these pathways is the mTOR signaling. mTOR is a highly conservative molecule in eukaryotic evolution, which is necessary for maintaining metabolic homeostasis, cell proliferation, survival, and promoting protein synthesis [8, 9]. By binding with other proteins, mTOR forms two different large complexes, mTOR complex 1 (mTORC1) and mTORC2, which show different serine/threonine kinase activities on their respective substrates. mTORC1 phosphorylates 4E-BP1 and p70S6K, while mTORC2 phosphorylates AKT at Ser473 [10]. In recent years, increasing studies have focused on the immunoregulatory function of mTOR signaling. For example, mTOR inhibitors have been reported to regulate immune responses, mainly serving as immunosuppressive agents [11, 12]. On the other hand, emerging evidence demonstrates that uptake of amino acids and hence activation of mTOR are required for gastrointestinal homeostasis against invasion of commensal microbes [13], suggesting mTOR's action on promoting innate immunity of the gut.

Peritoneal macrophages (pMΦs) are one type of tissue-resident macrophages that have been extensively explored, but their tissue-specific function has been poorly understood. One recent finding reveals that resident pMΦs are pivotal innate immune cells in maintaining gastrointestinal homeostasis [14]. At homeostasis, resident pMΦs are mainly composed of two populations: the large peritoneal macrophages (LPMs) with an F4/80^hi^MHCII^low^ phenotype predominate the peritoneal cavity, while the small peritoneal macrophages (SPMs) with an F4/80^low^MHCII^hi^ phenotype are the minor population. Although the developmental status of SPMs is largely unknown, the LPMs have been characterized as fetal-originated tissue-resident macrophages expressing a distinctive transcription factor GATA6 [15, 16], and regulating the function of peritoneal B-1 cells [14]. Thus, resident pMΦs under the steady-state conditions constitute a differing population from monocyte-derived macrophages (MDMs) of the peritoneal cavity in the inflammatory settings.

Although piperine has been shown to suppress innate immune responses in inflammatory MDMs from the peritoneal cavity [6], it is still unknown whether piperine has similar effects on resident pMΦs and whether its administration exacerbates or prevents peritoneal bacterial infection. In this study, we found that piperine potentiated the mTORC1 activity by recruiting system L1 amino acid transporter SLC7A5/SLC3A2 [17] to the cell membrane and promoting amino acid metabolism. Enhancement of the mTORC1 activity by piperine was correlated with reinforced functions of resident pMΦs upon bacterial stimulation, which conferred mice resistance against peritoneal bacterial infection and sepsis. Our data highlight piperine as a pharmacological booster of the mTORC1 activity during amino acid metabolism to enhance the innate functions of resident pMΦs against microbial infection.

## RESULTS

### Piperine enhances the mTORC1 activity depending on amino acid metabolism

The activity of piperine on the mTOR pathway was initially tested in cultured cell lines, and we discovered that piperine elevated the mTORC1 activity in human LNCaP and HeLa cells and mouse macrophage RAW 264.7 cells. Piperine treatment enhanced the phosphorylation of 4E-BP1 and p70S6K, two substrates of the mTORC1 kinase, indicating that this alkaloid could enhance the mTORC1 signaling in these cell lines (Supplementary Figure S1, A, B and C).

**Figure 1:**
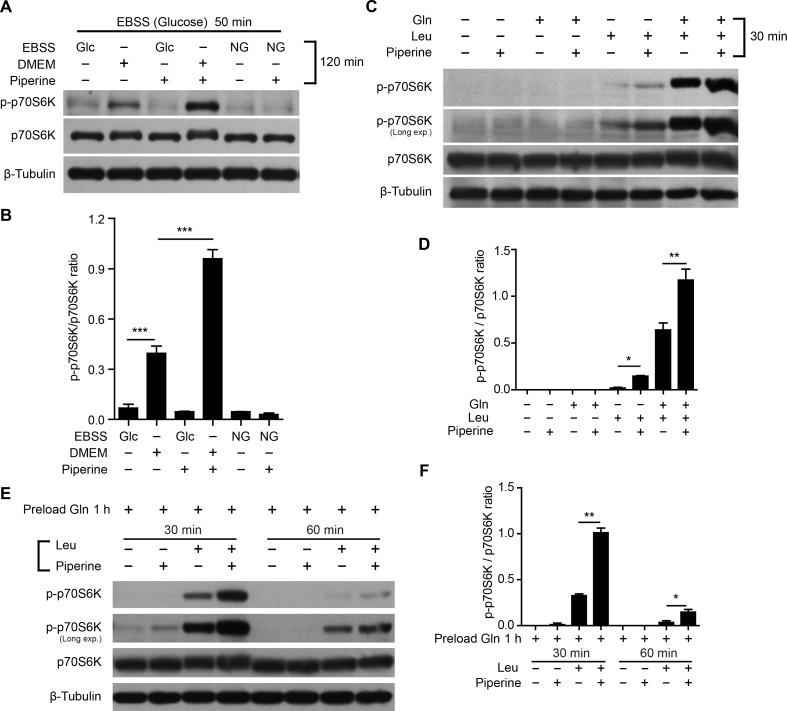
Piperine enhances mTORC1 activation in the presence of amino acids **A.**-**F.** Western blotting showing p-p70S6K levels in HeLa cells that were first starved in Earle's Balanced Salt Solution (EBSS, with glucose) for 50 min **A.** or Krebs-Ringer bicarbonate buffer (KRBB) for 50 min **C.** or KRBB for 3 h **E.**, followed by incubation in indicated media (serum-free) for indicated time periods. Gln (L-glutamine, 2 mM), Leu (L-leucine, 0.8 mM) and/or piperine (40 μM) were added in EBSS **A.** or KRBB **C.**, **E.**. Glc, glucose; NG, no glucose. Quantification of p-p70S6K relative to total p70S6K is shown in **B.**, **D.** and **F.** (*n = 3*). The significance was analyzed by Student's *t*-test. *P < 0.05; **P < 0.01; ***P < 0.001.

We next determined the action mechanism by which piperine enhanced the mTORC1 activity. mTOR is a central player in sensing nutrient sufficiency and regulating diverse metabolic processes [18]. It can be activated by hormones (e.g. insulin), nutrients, and cytokines [19]. Piperine has been shown to enhance the permeability of intestinal epithelial cells [20], increase the bioavailability of various drugs [21, 22] and promote the uptake of amino acids [20]. These studies prompted us to explore whether piperine enhanced the mTORC1 activity depending on nutrients in culture medium. To this end, HeLa cells were first starved in Earle's Balanced Salt Solution (EBSS) medium (without serum and amino acids) to suppress the basal mTORC1 activity, and then cultured in different media lacking serum, amino acids or glucose, respectively. The mTORC1 failed to be activated by EBSS medium, either with or without glucose. In contrast, the mTORC1 activity was elevated by serum-free DMEM, and was further potentiated by piperine co-treatment (Figure 1A and 1B).

Considering that DMEM contains amino acids but EBSS does not, these results suggested that piperine-induced elevation of the mTORC1 activity may rely on the presence of amino acids, instead of glucose. It is known that mTORC1 is activated during glutaminolysis, in which L-glutamine (Gln) is sequentially metabolized into L-glutamate and α-keroglutarate by glutaminase and glutamate dehydrogenase, respectively [23]. As an allosteric activator of glutamate dehydrogenase, L-leucine (Leu) promotes the conversion of L-glutamate to α-keroglutarate, which is a robust inducer of mTORC1 activation [24]. However, Gln is the rate-limiting amino acid for the mTORC1 activation by this way, since the efflux of intracellular Gln is required for the uptake of Leu and other essential amino acids via the bidirectional SLC7A5/SLC3A2 transporter [25]. Consistent with these studies, the mTORC1 activity was activated in HeLa cells in the presence of Leu, or Leu plus Gln, but not Gln alone, and combination of these amino acids with piperine further increased the mTORC1 activity (Figure 1C and 1D and Supplementary Figure S2A). Notably, after the cells were starved for a short period (50 min), which is likely insufficient to exhaust the intracellular Gln pool [23], piperine potentiated the mTORC1 activation induced by Leu treatment alone at 30 min (Figure 1C and 1D), but not at 2 h (Supplementary Figure S2A). Moreover, when Gln was preloaded into the cells having starved for 3 h, piperine markedly intensified Leu-induced mTORC1 activation at time point of 30 min (Figure 1E and 1F), and also enhanced it at earlier (Supplementary Figure S2B) or later time points (Figure 1E and 1F). Collectively, piperine-induced elevation of the mTORC1 activity relies on the uptake of specific (or essential) amino acids including Leu in the presence of intracellular Gln.

### Piperine enhances mTORC1 activity by recruiting amino acid transporters to the cell surface

**Figure 2:**
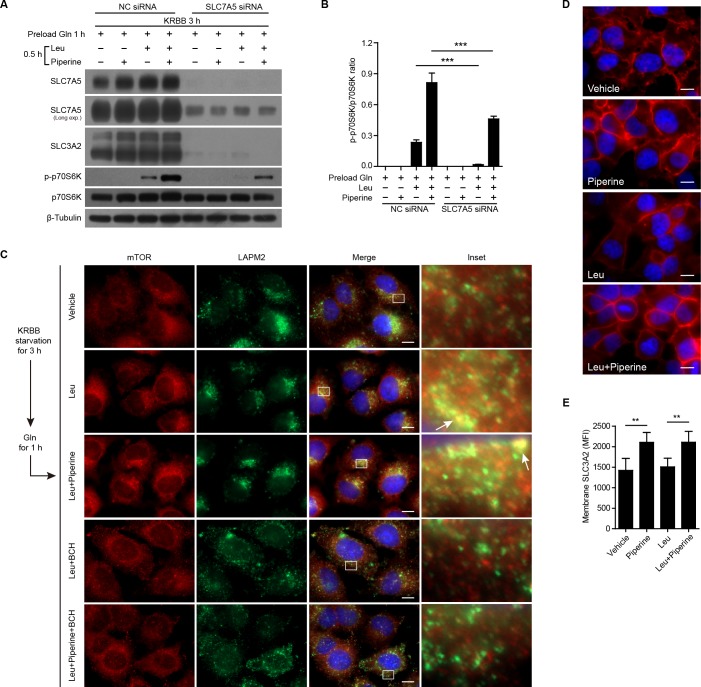
Piperine enhances Leu-induced mTOR activation through amino acid transporter **A.** SLC7A5 protein expression in HeLa cells was first knocked down with specific siRNA or negative control siRNA for 72 h. Afterwards, the cells were starved in KRBB and then sequentially loaded with Gln (2 mM), Leu (0.8 mM) and/or piperine (40 μM) for indicated periods. Western blotting was used to detect protein expression levels. The quantification of p-p70S6K relative to total p70S6K in **A.** is shown in **B.** as mean ± SD (*n = 3*). **C.** HeLa cells were first starved in KRBB and then sequentially loaded with Gln for 1 h, and Leu and/or piperine for 0.5 h. SLC7A5/SLC3A2 activity was inhibited by BCH (50 mM). Subcellular mTOR and LAMP2 distribution were revealed by immunofluorescence microscopy. Arrows indicate typical co-localization of mTOR and LAMP2 (yellow puncta). The nuclei (blue) were revealed by Hoechst33342 staining. **D.** HeLa cells were treated as **C.** and the subcellular distribution of SLC3A2 was revealed by immunofluorescence microscopy. **E.** Cell membranous SLC3A2 distribution was evaluated by its mean fluorescence intensity (MFI) (*n = 10* fields). Scale bars, 10 μm. The significance was estimated by Student's *t*-test. **P < 0.01; ***P < 0.001.

SLC7A5/SLC3A2 heterodimer is also known as system L1 amino acid transporter, the major cell surface transporter for Leu [17]. This is a bidirectional transporter as the export of Gln is required for the import of Leu [25], which can critically activates the mTORC1 activity [18, 25]. We explored whether the effect of piperine on mTORC1 activation was contributed by this bidirectional transporter. Consistent with previous reports [25], our result showed that knockdown of SCL7A5 with siRNA also downregulated the expression of SLC3A2 (Figure 2A and 2B). As expected, the effect of piperine on enhancing Leu-induced mTORC1 activation (as revealed by p70S6K phosphorylation) was greatly suppressed by SLC7A5 knockdown (Figure 2A and 2B). Likewise, immunofluorescence microscopy demonstrated that Leu induced the co-localization of mTOR and lysosome marker LAMP2, indicating the activation of mTOR on the lysosomal membrane. Once again, piperine treatment enhanced Leu-induced mTOR activation by promoting the translocation of mTOR onto the lysosome. However, when Leu transporter was blocked by 2-amino-2-norbornanecarboxylic acid (BCH), an inhibitor of SLC7A5 [25, 26], the co-localization of mTOR and LAMP2 was abrogated (Figure 2C). These results suggested that piperine enhanced Leu-induced mTORC1 activation through the system L1 amino acid transporter SLC7A5/SLC3A2. To further verify this hypothesis, the subcellular distribution of SLC7A5/SLC3A2 was observed by immunofluorescence microscopy analysis of the SLC3A2 subunit. The results showed that SLC3A2 was distributed at both cell membrane and intracellular compartments in vehicle-treated cells. After piperine treatment, regardless of the presence or absence of Leu, more SLC3A2 was distributed at the cell membrane, with increased mean fluorescent intensity (MFI) in this cellular compartment. However, the difference on cell-membranous MFI between vehicle and Leu groups was not statistically significant (Figure 2D and 2E). Thus, piperine appeared to poise cells to transport amino acids more efficiently by recruiting intracellular SLC7A5/SLC3A2 onto the cell membrane.

### Piperine treatment activates mTORC1 signaling in resident pMΦs

**Figure 3:**
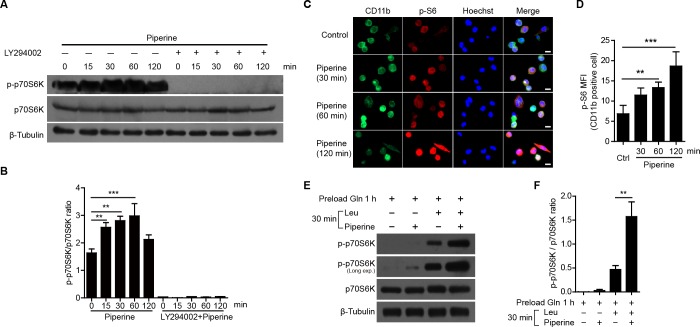
Piperine enhanced mTORC1 activation in mouse peritoneal resident macrophages **A.** Western blot analysis showing that piperine (40 μM) elevated the phosphorylation levels of p70S6K in mouse peritoneal resident macrophages, which was suppressed by LY294002 (10 μM) pretreatment. The quantification of p-p70S6K relative to total p70S6K in **A.** is shown in **B.** (*n = 3*). **C.** Immunofluorescence microscopy showing mTORC1 activity by the intracellular p-S6 levels (red) in CD11b^+^ (green) macrophages upon piperine exposure. The nuclei (blue) were revealed by Hoechst33342 staining. Scale bars, 10 μm. **D.** Quantitative analysis of p-S6 mean fluorescence intensity (MFI) in **C.** (*n = 10* fields). **E.** and **F.** Freshly isolated mouse peritoneal resident macrophages were starved in KRBB. Then the cells were preloaded with Gln, followed by piperine and Leu co-treatment as did in Figure 1E. The mTORC1 activation was detected by Western blotting **E.** and the quantification of p-p70S6K relative to total p70S6K is shown in **F.** (*n = 3*). The significance was estimated by Student's *t*-test. **P < 0.01; ***P < 0.001.

To confirm whether piperine potentiates mTORC1 activity in primary macrophages, we treated freshly isolated mouse resident pMΦs with piperine and measured the phosphorylation levels of p70S6K by Western blotting. Piperine significantly increased the phosphorylation levels of p70S6K, which could be attenuated by LY294002, an inhibitor of PI3K upstream of mTORC1 [27] (Figure 3A and 3B). Furthermore, immunofluorescence microscopy revealed that piperine time-dependently elevated the phosphorylation of S6 protein, a substrate of p70S6K, in CD11b^+^ resident pMΦs (Figure 3C and 3D). When resident pMΦs were preloaded with Gln after amino acid starvation for 3 h, piperine greatly enhanced mTORC1 activation in the presence of Leu (Figure 3E and 3F). Thus, piperine elevated the mTORC1 activity both in RAW 264.7 macrophage cell line and in primary resident pMΦs.

### Piperine treatment increases LPS-stimulated IL-6 and TNF-α expression in resident pMΦs, but not in thioglycollate (TG)-elicited ones

**Figure 4:**
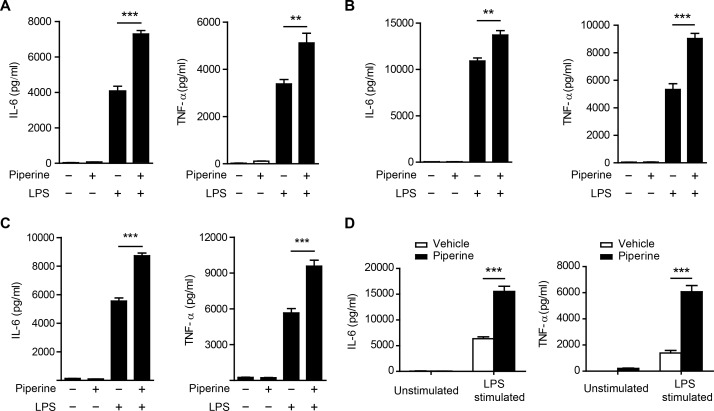
Piperine enhances cytokine expression in peritoneal resident macrophages upon LPS stimulation *in vitro* **A.**-**C.**, Mouse peritoneal resident macrophages were pretreated with or without piperine for 1 h **A.**, 6 h **B.**, or 48 h **C.**, followed by LPS (100 ng/ml) stimulation for 24 h. **D.** C57BL/6 mice were administered with piperine (20 mg/kg) or vehicle via gavage once a day for 10 consecutive days and their peritoneal resident macrophages were isolated, cultured *in vitro*, and stimulated with 100 ng/ml LPS for 24 h. Cytokines in the culture media were measured. All experiments were repeated for three times independently with one representative experiment presented in **A.**, **B.** and **C.** (mean ± SD, *n = 3*) and in **D.** (mean ± SD, *n = 5* mice). The significance was analyzed by Student's *t*-test. **P < 0.01; ***P < 0.001.

Although piperine has exhibited anti-inflammatory activity in different models [4-6], its effect on cytokine expression in resident pMΦs has not yet been evaluated. As enhanced mTOR activity can promote global protein synthesis [8-10] and increase IL-6 and TNF-α secretion in β-glucan-trained macrophages [28], we sought to explore whether piperine, which acted as a booster of mTORC1 activation, elevated the expression of these cytokines in resident pMΦs. Indeed, piperine pretreatment for either short periods (1 h and 6 h) or a prolonged period (48 h) markedly enhanced IL-6 and TNF-α production from resident pMΦs stimulated with LPS, while piperine alone had minimal effect (Figure 4A, 4B and 4C). Moreover, in response to LPS stimulation *in vitro*, resident pMΦs isolated from C57BL/6 mice which had intragastrically (i.g.) administered with piperine for 10 days produced more than two-fold amounts of IL-6 and TNF-α as much as that of their counterparts from vehicle-treated mice. But without LPS stimulation, the expression of these cytokines was low in the pMΦs isolated from both piperine-conditioned and control mice (Figure 4D).

However, when thioglycollate (TG)-elicited pMΦs were used for experiments, piperine pretreatment did not change IL-6 and TNF-α expression upon LPS stimulation (Supplementary Figure S3A), but even suppressed the expression of interferon-γ (Supplementary Figure S3B), in step with a previous study [6]. As aforementioned, pMΦs may be classified into two populations based on their expression of macrophage markers F4/80 and MHC class II (MHCII) molecules [14]. The differential responses of cytokine expression in resident pMΦs and TG-elicited ones is likely because they are different types of macrophages, with the latter being mostly MDMs as a result of TG stimulation.

To confirm this, we analyzed the phenotypes of these two macrophage populations by flow cytometry. TG treatment suppressed the expression of F4/80 in the pMΦs, while the resident pMΦs in control group contained more LPMs but fewer SPMs as compared to TG-elicited cells (Supplementary Figure S4A). Moreover, GATA6, a transcription factor critical for resident pMΦ function and survival [16], was highly expressed in resident pMΦs, but only in a minor subset of TG-elicited cells (Supplementary Figure S4A and S4B). In addition, TG-elicited pMΦs were, on average, larger in size than the resident ones (Supplementary Figure S4B). Therefore, the resident and TG-elicited pMΦs were composed of different cell populations, thus representing different types of macrophages, which may probably account for their differential patterns of cytokine expression upon piperine treatment.

### Piperine-induced enhancement of cytokine production is abrogated by PI3K/AKT/mTOR pathway inhibitors

**Figure 5:**
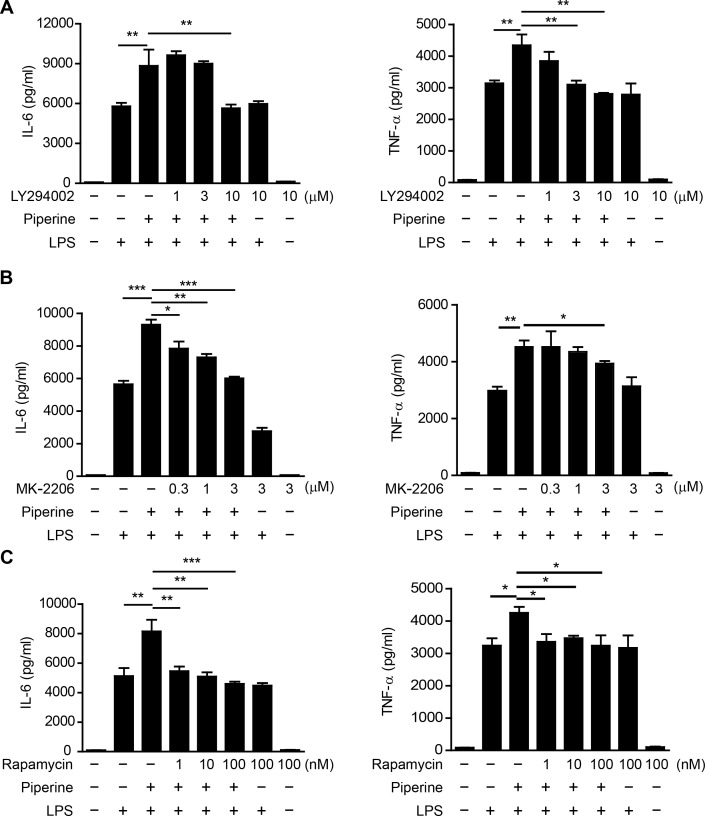
Piperine enhances IL-6 and TNF-α expression in peritoneal resident macrophages in an mTOR-dependent manner Isolated mouse peritoneal resident macrophages were pretreated with indicated concentrations of PI3K inhibitor LY294002, AKT inhibitor MK-2206 or mTOR inhibitor rapamycin for 30 min. Piperine (40 μM) were added to the media for 1 h and the cells were stimulated with 100 ng/ml LPS for 24 h. Cytokines in culture media were measured. All experiments were repeated for three times independently with one representative experiment presented in **A.**, **B.** and **C.** (mean ± SD, *n = 3*). The significance was estimated by one-way ANOVA followed by Tukey's multiple comparison test. *P < 0.05; **P < 0.01; ***P < 0.001.

As the above data indicated that piperine elevated the mTORC1 activity concomitant with enhanced cytokine expression in resident pMΦs, we next explored the causality between these two processes by blocking mTOR activation during piperine treatment. Both indirect mTORC1 inhibitors LY294002 (PI3K inhibitor) [27] and MK-2206 (AKT inhibitor) [29], and direct inhibitor rapamycin [30], completely abrogated the enhancement of IL-6 and TNF-α expression in the presence of piperine. However, they had minimal effect on LPS-induced cytokine expression except MK-2206's effect on IL-6 expression (Figure 5A, 5B and 5C). These results indicated that piperine increased IL-6 and TNF-α protein expression by enhancing the mTOR signaling.

### Piperine administration provides protection against bacterial infection and sepsis

**Figure 6:**
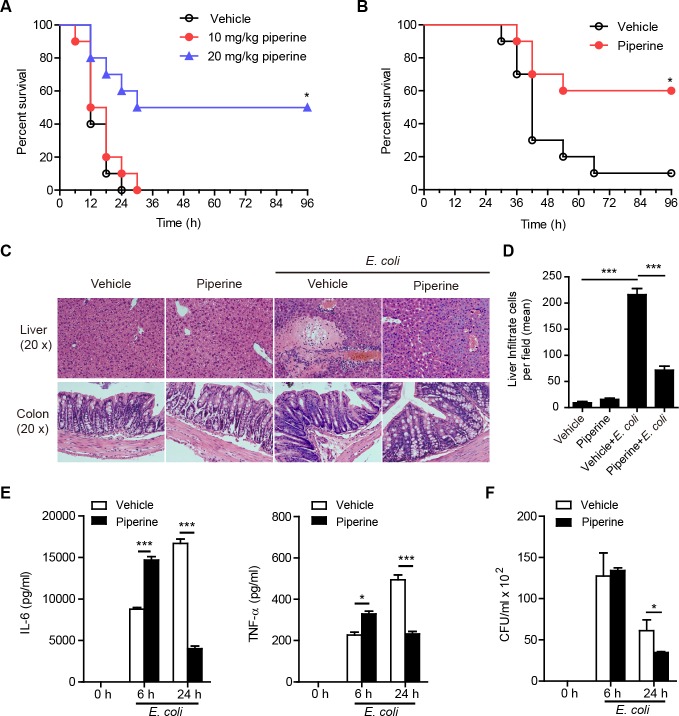
Piperine administration confers resistance to bacterial infection and attenuates sepsis **A.** and **B.** Survival of C57BL/6 mice infected with viable *E. coli*. Mice (*n = 10* for each group) were administered with piperine intragastrically prior to **A.** or after (**B.**; piperine 20 mg/kg) bacterial inoculation as described in Methods. The significance was evaluated by the nonparametric Mann-Whitney *U* test. *P < 0.05. **C.** Representative haemotoxylin and eosin staining of the liver and colon 8 h after infection from C57BL/6 mice (*n = 5* for each group) treated as in **A.**. **D.** Quantification of infiltrate cells in the liver. (E and F) IL-6 and TNF-α **E.** and bacterial counts **F.** in the peritoneal cavity of *E. coli*-infected mice. C57BL/6 mice (C-F) were treated with or without piperine (20 mg/kg) as in **A.**, followed by *E. coli* (1×10^9^ CFU/mouse) infection. The experiments were repeated for three times independently with one representative experiment presented (mean ± SD, *n = 5*). Bacterial counts were measured by LB agar culture **F.**. The significance was estimated by Student's *t*-test. *P < 0.05; **P < 0.01; ***P < 0.001.

As IL-6 and TNF-α are critical for pathogen clearance by enhancing the functions of phagocytes [31, 32] and by promoting the expression of antimicrobial peptides [33], we next further assessed whether enhanced IL-6 and TNF-α production in resident pMΦs of mice having administered with piperine conferred resistance to bacterial infection and septic shock. To verify this, C57BL/6 mice were infected with lethal or sub-lethal doses of viable *Escherichia coli*, and piperine was administered before (prophylactic model) or after (therapeutic model) bacterial inoculation. The results showed that piperine treatment significantly protected mice against lethal bacterial infection either in the prophylactic (Figure 6A) or therapeutic model (Figure 6B). For the prophylactic model, the control mice that were infected with a lethal dose of viable *E. coli* (2×10^9^ CFU/mouse) into their peritoneal cavity died within 24 h, but those having administered with piperine had higher survival rates after bacterial infection. Among the mice having treated with 20 mg/kg but not 10 mg/kg piperine, 60% survived the first 24 h after bacterial challenge and 50% were still alive in an active state after 96 h (*P* < 0.05; Figure 6A). In the therapeutic model, piperine (20 mg/kg) treatment 1 h after bacterial infection with a sub-lethal dose of *E. coli* (1×10^9^ CFU/mouse) also reduced the mouse mortality (*P* < 0.05; Figure 6B). Histochemical analysis demonstrated that viable bacterial infection in the peritoneal cavity had induced sepsis, but the symptoms of sepsis were alleviated by piperine treatment, with fewer infiltrated leukocytes in the liver tissue sections and no obvious inflammation in the colon (Figure 6C and 6D).

As expected, bacterial infection greatly increased the expression of IL-6 and TNF-α in their peritoneal cavity, whereas such cytokine levels were lower or undetectable without bacterial infection. Interestingly, compared with controls, piperine-administered mice had significant higher IL-6 and TNF-α levels in their peritoneal cavity lavage fluids at 6 h but had lowered levels of these cytokines at 24 h (Figure 6E) after bacterial infection, accompanied with a declined bacterial burden at 24 h (Figure 6F). Therefore, piperine administration to mice appeared to enhance the innate functions of their pMΦs, thereby accelerating bacterial clearance and inflammation resolution in their peritoneal cavity.

### Piperine protects resident pMΦs from bacterium-induced apoptosis

**Figure 7:**
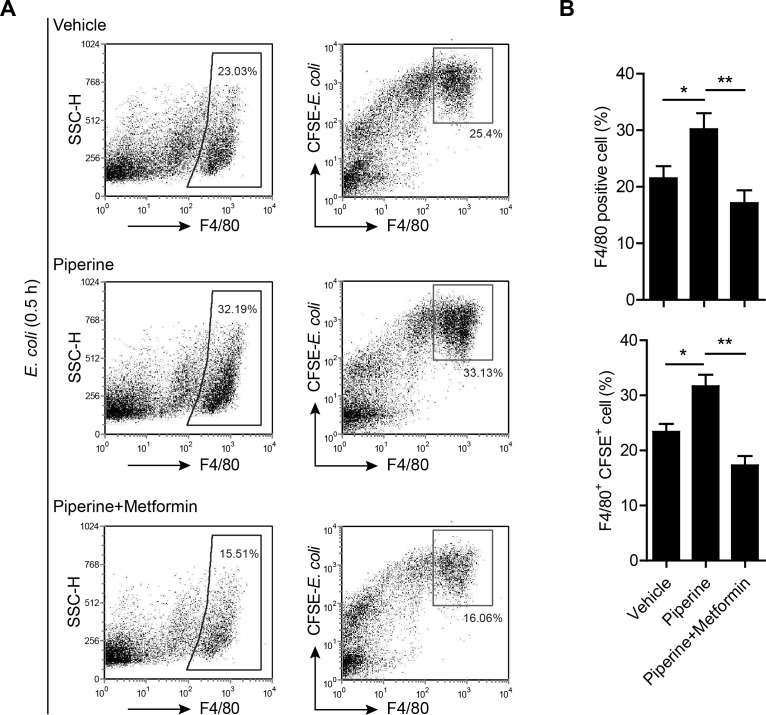
Resident pMΦs from piperine-treated mice exhibits increased capacity to phagocytize bacteria **A.** Mice were administered with piperine (20 mg/kg) or piperine plus metformin (250 mg/kg) intragastrically once and 4 h later were injected with CFSE-labeled *E. coli* (equivalent to 0.5×10^9^ CFU viable bacteria/mouse) intraperitoneally for 0.5 h. Afterwards, the peritoneal cells were isolated and stained with F4/80 antibody and analyzed by flow cytometry. Data were from one representative set of 3 independent experiments. **B.** Quantitative analyses of percentages of F4/80^+^ macrophages and percentages of F4/80^+^CFSE^+^ cells in gated cells. Data are expressed as mean ± SD (*n = 5*). The significance was estimated by one-way ANOVA followed by Tukey's multiple comparison test. **P < 0.01; ***P < 0.001.

The lowered bacterial burden in piperine-administered mice prompted us to explore whether piperine treatment had enhanced the bacterial phagocytic ability of their resident pMΦs. For this purpose, mice were injected with CFSE-labeled bacteria and their resident pMΦs were analyzed by flow cytometry. The results showed that most F4/80^hi^ cells also had highest CFSE intensity (Figure 7A), suggesting that the F4/80^hi^ resident pMΦs were the major bacterial phagocytic cells at the early stage of infection, which is in step with previous reports [34]. It was noticeable that the ratio of F4/80^hi^ resident pMΦs in piperine-administered group was higher than that of control (vehicle group) (Figure 7), suggesting that piperine treatment protected the F4/80^hi^ resident pMΦs from bacterium-induced disappearance, a phenomenon having been observed in peritonitis decades ago [35]. However, co-treatment with metformin, an activator of AMPK and indirect inhibitor of mTOR signaling [28], greatly attenuated such an effect of piperine, reducing both the ratios of F4/80^hi^ and F4/80^hi^CFSE^+^ pMΦs (Figure 7).

**Figure 8:**
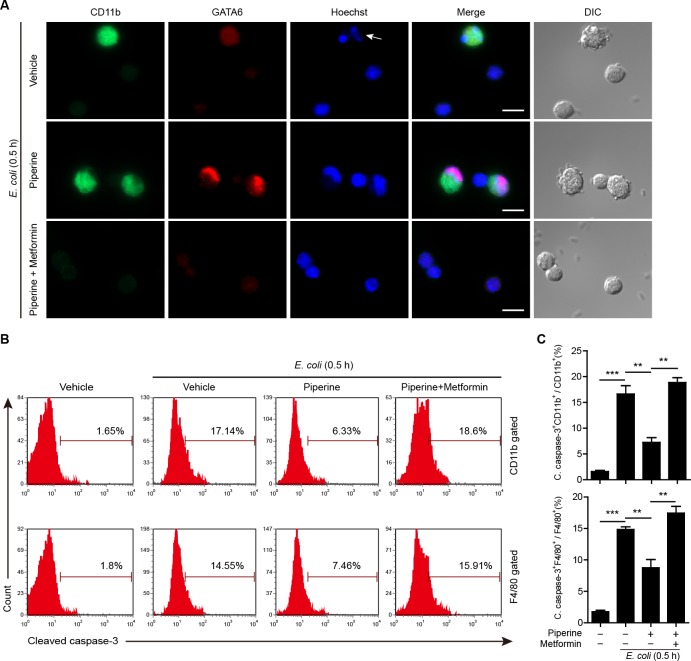
Piperine protects resident peritoneal macrophages from bacterium-induced apoptosis **A.** Mice (*n = 5* for each group) were administered with piperine (20 mg/kg) or piperine plus metformin (250 mg/kg) intragastrically once and 4 h later were injected with viable *E. coli* (0.5×10^9^ CFU/mouse) intraperitoneally for 0.5 h. The peritoneal cells were isolated and seeded in glass-bottom culture dishes. After washing, the macrophages were fixed and stained with CD11b (green), GATA6 (red) and Hoechst 33342 (blue). Images were captured by fluorescence microscopy. DIC images are also shown. The arrow indicates karyorrhexis. Scale bars, 10 μm. **B.** Mice were treated as in **A.** and their peritoneal macrophages were isolated and stained with CD11b, F4/80 antibody, followed by intracellular staining with cleaved caspase-3 antibody. One representative flow cytometry plots of 3 independent experiments are shown. **C.** Quantitative analysis of the percentages of cleaved caspase-3 positive cells in CD11b^+^ or F4/80^+^ macrophages. Data are expressed as mean ± SD (*n = 5*). The significance was estimated by Student's *t*-test. **P < 0.01; ***P < 0.001.

These data indicated that fewer pMΦs could be isolated from the peritoneal cavity of mice infected with bacteria as compared with control mice. Indeed, the F4/80^hi^ cells in normal mice accounted for about 80% of the total pMΦs (Supplementary Figure 4A), but after infection this ratio could be reduced below 30% (Figure 7). It appeared that bacterial infection had quickly (within 0.5 h) led to resident pMΦ disappearing. However, piperine treatment seemed to attenuate the disappearance of F4/80^hi^ pMΦs by bacterial infection (Figure 7). Considering the effect of piperine on mTOR activation and the role of mTOR in cell survival [36], we explored whether piperine protected F4/80^hi^ resident pMΦs from cell death. To this end, mice were infected with viable bacteria for 0.5 h and their pMΦs were immediately isolated and seeded in glass-bottom culture dishes, followed by CD11b and GATA6 antibody staining. It has been reported that GATA6 expression is a characteristic for resident pMΦs, which is vital not only for their role in regulating peritoneal B-1 cells [14], but also for their own renewal and survival [15, 16]. As shown in Figure 8A, many bacterium-containing vacuoles could be observed in CD11b^hi^/GATA6^bright^ pMΦs, but not in CD11b^low^/GATA6^faint^ cells. In addition, over-phagocytosis of viable bacteria might have led to apoptosis (as judged by karyorrhexis) of the former cells, instead of the latter ones, and GATA6 was diffusely distributed in the apoptotic cells. Piperine pre-administration prevented the CD11b^hi^/GATA6^bright^ pMΦs from apoptosis, and confined GATA6 within their nuclei. Of note, few CD11b^hi^/GATA6^bright^ pMΦs could be observed in either the vehicle or piperine+metformin group, possibly because this cell population had died from infection and thus detached from dishes.

To quantitatively evaluate cell apoptosis, mice were infected with viable *E. coli* and their pMΦs were analyzed after intracellular cleaved caspase-3 staining. Caspase-3 is a critical executive protease in apoptosis process [37]. As shown in Figure 8B and 8C, bacterial infection increased cleaved caspase-3 levels in macrophages, suggesting that apoptosis was initiated early (0.5 h post infection) after phagocytosis of bacteria. Piperine treatment reduced the cleaved caspase-3 levels in both CD11b^+^ and F4/80^+^ pMΦs, but this effect of piperine could be blocked by metformin treatment. Together, these results suggested that piperine treatment (thus enhancing the mTOR activity) protected resident pMΦs from bacterium-induced apoptotic cell death.

## DISCUSSION

Peritoneal bacterial infection or even bacterial sepsis, due to the penetration of intestinal bacteria, constitutes a great challenge in the treatment of bowel diseases. Here we reported that piperine enhanced the mTORC1 activity by recruiting amino acid transporter to the cell membrane and promoting amino acid metabolism (e.g., glutaminolysis). Such an activity of piperine was linked to its anti-bacterial action at least in resident pMΦs of mice. The prophylactic and therapeutic model study demonstrated that piperine were effective for preventing bacterial infection and even for sepsis. To our knowledge, we for the first time reported that a small molecule alkaloid has the capacity to enhance mTORC1 activity and thus fortifies the functions of resident pMΦs.

It has long been established that piperine increases bioavailability of clinical drugs [21, 22], accelerating uptake of amino acids (including Leu) and other nutrients by intestinal epithelial cells [20]. But the mechanism underlying this process is unclear. It was unlikely that Leu could pass through the cell membrane in a non-specific manner, since it is a large neutral (aliphatic) amino acid relying on distinct transporters for its uptake into cells. Our study provided evidence showing that piperine promoted amino acid metabolism by recruiting more SLC7A5/SLC3A2, the system L1 amino acid transporter for Leu and other large neutral ones, to the cell membrane, which in turn enhanced mTORC1 activation [18, 25]. Previous reports have indicated that mTOR is a central player in sensing nutrient sufficiency and regulating diverse metabolic processes [18]. Uptake of amino acids, such as Leu and Gln, leads to mTORC1 activation on the lysosomal surface, in a Rag small guanosine triphosphatases (GTPases)-dependent or independent manner [23, 38, 39]. Amino acid metabolism and mTORC1 activation may rely on the cell membrane abundance of the transporter, instead of its total cellular protein levels. Supporting this, although the total SLC7A5/SLC3A2 protein levels were greatly suppressed by SLC7A5 knockdown, the mTORC1 activity in SLC7A5-knocked down cells co-treated with piperine and Leu was still higher than that in control cells treated with Leu alone (Figure 2A and Figure 2B). The uptake of Leu by SLC7A5 has been reported to be required for the metabolic reprograming (mTOR activation) essential for T cell differentiation in response to antigens [40]. At certain circumstances, such as hepatitis B virus infection [41], T cells may upregulate SLC7A5 expression to meet the requirement for Leu and metabolic reprograming. Thus, likely acting through a similar mechanism, piperine pharmacologically potentiates mTORC1 activation by reprogramming amino acid metabolism.

Our study demonstrated that increased mTORC1 activation by piperine enhanced the functions of resident pMΦs, including their bacterial phagocytic ability and LPS-induced cytokine-producing capacity. Consistent with the role of mTOR signaling in cell survival [36], our results also demonstrated that piperine administration prevented F4/80^hi^ resident pMΦs from apoptosis. Due to such functional enhancements of resident pMΦs, piperine enhanced bacterial clearance and protected mice against bacterial infection in the peritoneal cavity. Therefore, the mTORC1-upregulatory capacity of piperine is of clinical significance: it may provide protection against infection for humans in a state of sub-malnutrition (in which the mTORC1 activity is below normal levels), such as in the case of inflammatory bowel disease (IBD) [13, 42]. In support of this hypothesis, uptake of amino acids and hence activation of mTOR are required for the maintenance of intestinal homeostasis [13], whereas malnutrition or suppression of mTOR activity induces colitis due to dysregulated innate immune functions of the intestine [43].

In recent years, many studies focused on the immunoregulatory action of mTOR signaling in macrophages [44, 45]. It has been reported that the fungal cell wall component β-glucan can prime monocytes/macrophages to mount an enhanced innate response against fungal infection by activating the mTOR pathway. One characteristic of β-glucan-primed monocytes is their enhanced ability to secrete IL-6 and TNF-α upon pathogen stimulation [28]; the authors named such immunological memory traits of innate immune cells as “trained immunity”. Consistent with this finding, our data demonstrated that enhanced mTORC1 activity in piperine-pretreated cells was linked to increased IL-6 and TNF-α expression and enhanced phagocytic capacity of resident pMΦs, as blocking the mTOR pathway by specific inhibitors attenuated all these effects of piperine. These results suggest that piperine and β-glucan may act through certain common mechanisms for enhancing the innate functions of monocytes/macrophages to prevent pathogen infection, but further investigation is required to clarify this issue.

It has been known that IL-6 and TNF-α secretion by macrophages upon bacterial infection in turn enhances their phagocytic capacity, thus promoting the clearance of pathogens [31, 46, 47]. Indeed, increased IL-6 and TNF-α production was correlated with enhanced bacterial clearance in piperine-administered mice compared to control mice (Figure 6F). Immunofluorescence observation demonstrated that it was the LPMs (CD11b^bright^GATA6^bright^), instead of SPMs, robustly engulfed bacteria (Figure 8A). These results demonstrated that the resident pMΦs were the major bacterial phagocytic cells at least at the early stage of infection and that their phagocytic capacity was enhanced by piperine treatment. In light of the notion that IL-6 and TNF-α promote wound healing and tissue repair [48-50], increased production of these cytokines may also limit pathogens from penetrating the epithelial lining of the intestine and invading into the peritoneal cavity to induce sepsis, an extreme case of IBD [51].

In summary, we demonstrated that piperine promoted amino acid metabolism and thus enhanced the mTORC1 activation in peritoneal resident macrophages, resulting in potentiation of their innate functions against bacterial infection, alleviating bacterial septic shock. Our study highlights the value of piperine not only as a major ingredient in daily seasoning for promoting appetite and digestion, but also as an mTORC1 booster to enhance the innate immunity of tissue-resident macrophages in fighting against diverse pathogens in certain clinical settings.

## MATERIALS AND METHODS

### Reagents and animals

LPS (*E. coli* 0111:B4), dimethyl sulfoxide (DMSO), Tween-80, Hoechst 33342 and amino acid transporter inhibitor BCH (2-amino-2-norbornanecarboxylic acid) were bought from Sigma-Aldrich (St. Louis, MO, USA). Thioglycollate medium (Brewer modified) was obtained from Becton Dickinson (Sparks, MD, USA). Piperine was purchased from Guangzhou Institute for Drug Control (Guangzhou, China), dissolved in DMSO and stored at −20°C. Rabbit antibodies against phospho(p)-p70S6K, p70S6K, p-4E-BP1, 4E-BP1, p-S6(Ser235/236), GATA6, cleaved caspase-3, SLC7A5, SLC3A2, mTOR, and β-tubulin were purchased from Cell Signaling Technology (Danvers, MA, USA). The mouse antibody against LAMP2 was obtained from Abcam (Cambridge, MA, USA). PE-F4/80, FITC-CD11b, AlexaFluor488-CD11b, and APC-MHCII were obtained from eBioscience (San Diego, CA, USA). DMEM medium, fetal bovine serum (FBS), penicillin and streptomycin were products of Invitrogen (Carlsbad, CA, USA).

Female C57BL/6 mice were bought from the Experimental Animal Center of Southern Medical University (Guangzhou, China). Animal experiments were designed following National Institutes of Health guidelines and were approved by the Committee on the Ethics of Animal Experiments of Jinan University.

### Bacterial preparation

*E. coli* strain DH5α were grown in Luria Broth (LB) media and shaken overnight at 37°C, and then re-inoculated into fresh LB at 10% for 3 h at 37°C. Bacteria cells were pelleted by centrifugation at 1824 ×g for 10 min, washed with 10 ml PBS twice, and resuspended in appropriate volume of PBS. Cell density was determined using an ultraviolet-visible spectrophotometer (Nanodrop 2000; Thermo Scientific) and the corresponding colony-forming units (CFU) were determined on LB agar plates. For some experiments, viable *E. coli* cells were stained with 40 μM carboxyfluorescein diacetate, succinimidyl ester (CFSE), washed and then inactivated for 1 h at 70°C in water bath.

### Bacterial infection

Mice were acclimated for one week. Viable *E. coli* DH5α resuspended in 0.5 ml of PBS was injected into the peritoneal cavity (i.p.), and the lethal (2×10^9^ CFU) and sub-lethal doses (1×10^9^ CFU) were determined by preliminary experiments, which induced peritonitis or even severe sepsis. Piperine (dissolved in 2% Tween-80 in PBS as a suspension) or vehicle (2% Tween-80 in PBS) was given intragastrically (i.g.) before or after bacterial infection. In the former model, mice were administered with piperine at a dose of 10 or 20 mg/kg once a day for 3 d consecutively prior to bacterial infection (2×10^9^ CFU); in the latter model, mice were infected with bacteria (1×10^9^ CFU) and 1 h later were given with 20 mg/kg piperine for only once. Mouse survival was monitored every 6 h for 4 d. In another experiment, the bacterial burden in the peritoneal cavity of mice was determined. The mice were sacrificed and peritoneal lavage fluids were collected with 1.5 ml PBS. Serial dilutions were made of the lavage fluids and then incubated overnight at 37°C on LB agar plates; CFUs of bacteria were counted and calculated.

### Peritoneal macrophage isolation

For *in vitro* study, mice were sacrificed by cervical dislocation and sterilized by 70% ethanol. Peritoneal macrophages were immediately isolated by washing the peritoneal cavity with washing buffer (sterile PBS containing 5% newborn calf serum and 0.5 mM EDTA). The extracted solution was centrifuged at 300 ×g for 10 min and isolated cells were cultured at 37°C in DMEM plus 10% FBS, 100 U/ml penicillin and 100 μg/ml streptomycin (complete medium). After 2-h incubation, unattached cells were discarded and attached macrophages were further cultured in fresh complete medium. In some experiments, C57BL/6 mice were first injected via i.p. with 1 ml of 3% thioglycollate (TG). Peritoneal macrophages were isolated 4 d post the injection.

### Cell line culture

Human cervical cancer HeLa cells, prostate cancer LNCaP cells and mouse macrophage cell line RAW 264.7 was obtained from the Cell Bank of the Chinese Academy of Sciences (Shanghai, China). Cells were maintained in complete DMEM medium and were cultured at 37°C in a humidified incubator with 5% CO_2_.

### Determination of soluble cytokines

Proinflammatory cytokine proteins in cell culture medium or mouse peritoneal cavity lavage fluids were determined by Cytometric Bead Array (CBA) mouse inflammation kit (BD Biosciences; San Jose, CA, USA) according to the manufacturer's instructions.

### Western blotting

HeLa, LNCaP, and RAW 264.7 cells were seeded in plastic flasks and cultured for 24 h and then changed with new culture medium with indicated doses of piperine. Whole cell lysates and Western blotting were performed as previously described [11].

### Immunofluorescence microscopy

Immunofluorescent staining was performed essentially as previously described [52]. Briefly, peritoneal macrophages were cultured in glass-bottom dishes, fixed, permeabilized and immunostained with rabbit anti-p-S6 antibody or anti-GATA6 antibody and AlexaFluor488-CD11b, followed by CF568-conjugated goat-anti-rabbit IgG (Biotium, Hayward, CA, USA). HeLa cells were immunostained with rabbit anti-mTOR antibody and mouse anti-LAMP2 antibody, or anti-SLC3A2 antibody alone, followed by CF488A-conjugated goat-anti-mouse IgG (Biotium) and/or CF568-conjugated goat-anti-rabbit IgG. Nuclei were revealed by Hoechst 33342 staining. Cells were observed using a Zeiss Axio Observer D1 microscope with a Zeiss EC Plan-Neofluar 100×/1.30 Oil M27 objective (Carl Zeiss MicroImaging GmbH, Göttingen, Germany). Fluorescence images were captured with a Zeiss AxioCam MR R3 cooled CCD camera controlled with ZEN software (ZEISS).

### Histopathology

After mice were sacrificed, liver and small intestine were harvested, fixed in 4% neutral formaldehyde, and stained with haemotoxylin and eosin (H&E). Images were captured under a microscope armed with a color CCD (Zeiss Axio Observer D1).

### Knockdown of SLC7A5

HeLa were seeded in 6-well plates (Corning) (for Western blotting) or glass bottom culture dishes (NEST, China) (for immunofluorescent staining) for 24 h. SLC7A5 siRNAs and negative control were produced by Ribobio (Guangzhou, China). The SLC7A5-specific siRNA sequence is 5′-GCAUCGGCUUCACCAUCAUdTdT-3′ targeting 5′-GCATCGGCTTCACCATCAT-3′. Knockdown of SLC7A5 was performed using Lipofectamine RNAiMAX (Invitrogen) according to the instructions provided by the manufacturer. The siRNA was added to each well at a final siRNA concentration of 100 nM. Six hours later, cells were cultured in normal medium containing 10% FBS. After being cultured for another 72 h, cells were used for mTOR activation experiment with Gln, Leu and/or piperine.

### Flow cytometry

For determination cell phenotypes or intracellular protein expression, cells were stained with antibodies, respectively, according to the manufacturers' instructions. CBA beads or cells were analyzed on a flow cytometer (FACSCalibur; Becton Dickinson, Mountain View, CA, USA), and data were acquired using the CELLQuest software (Becton Dickinson).

### Statistical analysis

All experiments were performed independently at least three times, with one representative experiment shown. Data are presented as mean ± standard deviation (SD). Statistical analysis was performed using Graphpad Prism 4.0 (GraphPad; San Diego, CA). One-way analysis of variance (ANOVA) followed by Tukey's multiple comparison test and unpaired Student's *t* test (2 tailed) were used to analyze the statistical significance among multiple groups and between two groups, respectively. Kaplan-Meier survival curves were adopted for analysis of mice survival, and the statistical difference between 2 groups was determined using the nonparametric Mann-Whitney *U* test. *P* values < 0.05 were considered statistically significant.

## SUPPLEMENTARY MATERIAL FIGURES


