# Supplementary material for: Piperine metabolically regulates peritoneal resident macrophages to potentiate their functions against bacterial infection
Source: Oncotarget. 2015 Oct 2;6(32):32468–83. doi: 10.18632/oncotarget.5957 (PMC4741706; doi:10.18632/oncotarget.5957)
Supplement: Supplementary file 1 [file oncotarget-06-32468-s001.pdf]

Piperine metabolically regulates peritoneal resident macrophages to potentiate their functions against bacterial infection

Supplementary Material

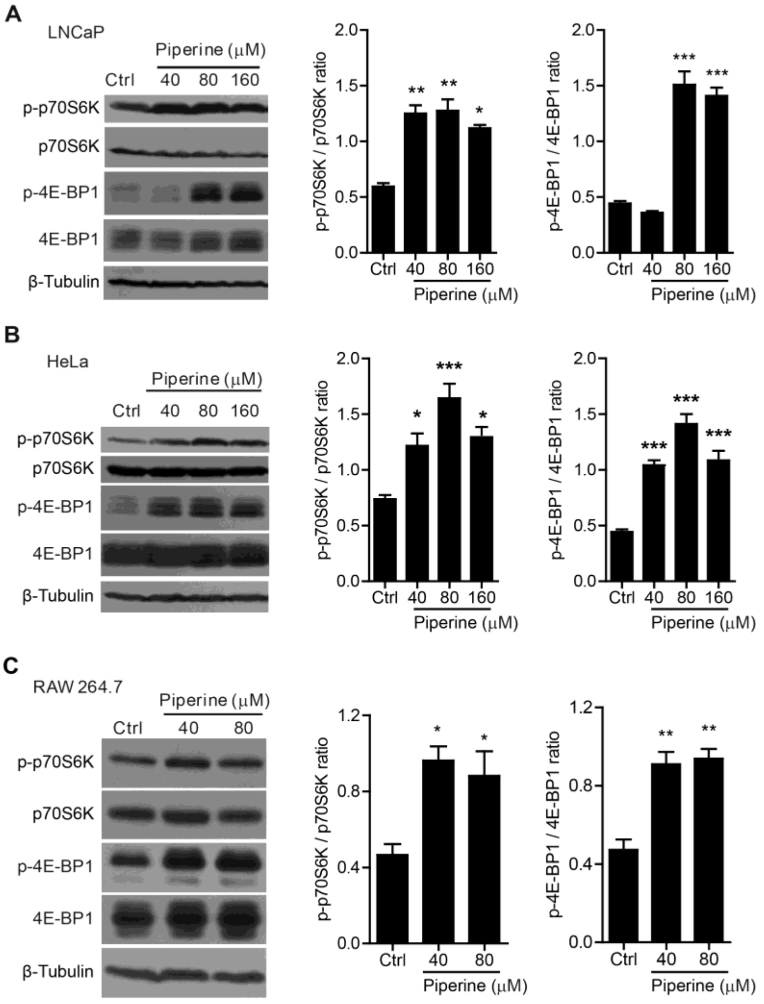

Supplementary Figure S1: Piperine enhances mTOR activity in cultured cell lines. Human LNCaP cells (**A**), HeLa cells (**B**) and mouse RAW 264.7 cells (**C**) were treated with indicated concentrations of piperine for 2 h. Western blot analysis was used to detect the phosphorylation of p70S6K and 4E-BP1 in these cells. Quantitative analysis of the ratios of phospho(p)-p70S6K to total p70S6K, and p-4E-BP1 to total 4E-BP1 in these cells are shown in the right panels, respectively. Data are presented as mean ± SD (*n*=*3*). The significance was estimated by Student's *t*-test. **P* < 0.05; ***P* < 0.01; ****P* < 0.001.

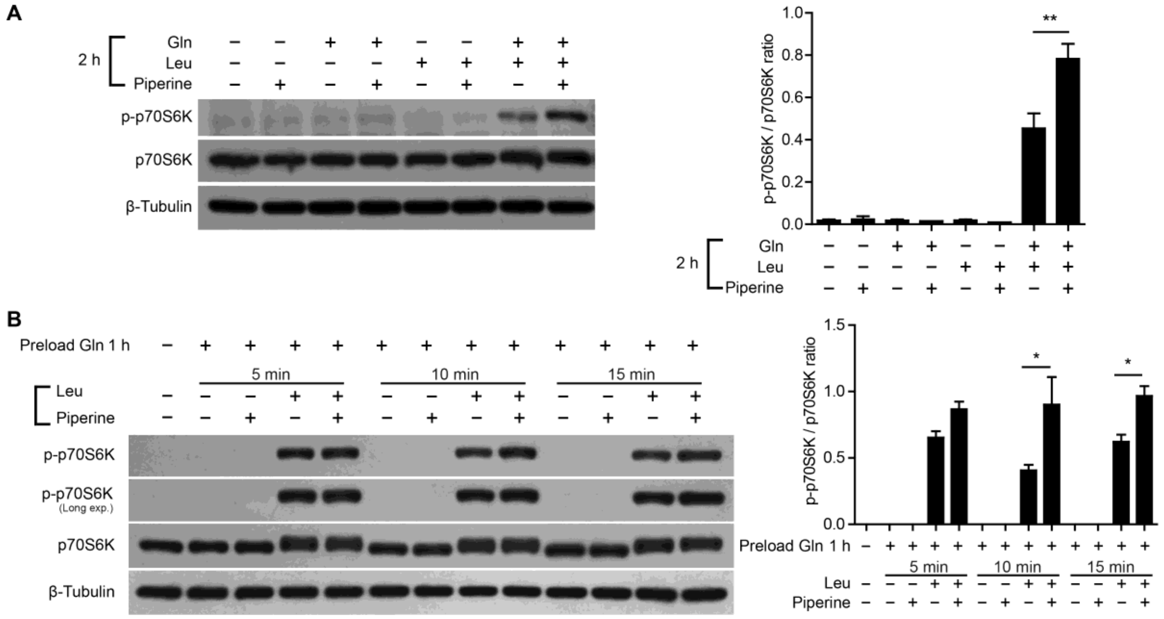

Supplementary Figure S2: Piperine potentiates mTOR activation during amino acid metabolism. (A) HeLa cells were starved in KRBB for 50 min and then incubated in KRBB medium with L-glutamine (Gln), L-leucine (Leu) and/or piperine (40 μM) for 2 h. (**B**) Cells were starved in KRBB for 3 h and then incubated with Leu and/or piperine for indicated time lengths. Western blotting was used to detect the phosphorylation levels of p70S6K. p70S6K activation was analyzed by the ratios of p-p70S6K to total p70S6K protein. Data are presented as mean ± SD (*n=3*). The significance was estimated by Student's *t*-test. *$P < 0.05$; **$P < 0.01$.

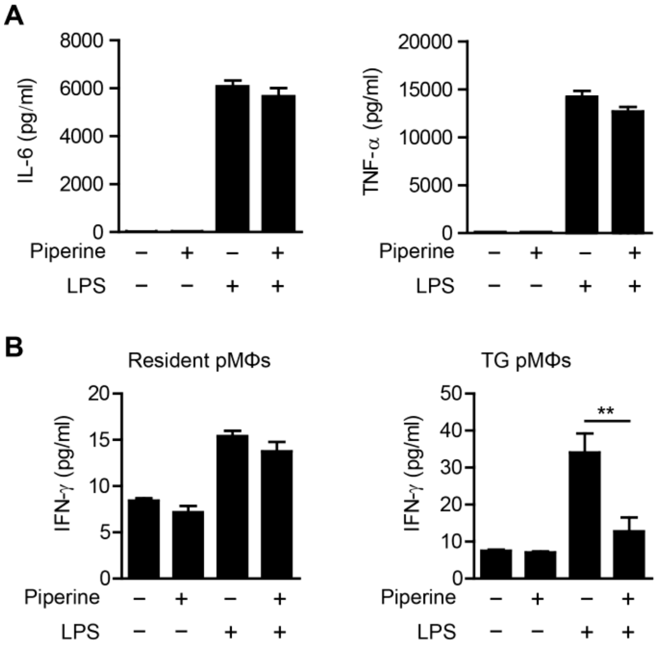

Supplementary Figure S3: Piperine exhibits differential effects on cytokine expression in peritoneal resident and thioglycollate (TG)-elicited macrophages stimulated with LPS. (A) IL-6 and TNF-α expression in TG-elicited macrophages. C57BL/6 mice were injected i.p. with 1 ml PBS containing 3% TG medium. Four days later, peritoneal macrophages (pMΦs) were isolated and cultured in 24-well plates. The cells were incubated with piperine for 48 h and then stimulated with 100 ng/ml LPS for 24 h. **(B)** IFN-γ expression upon LPS stimulation for 24 h *in vitro* in resident and TG-elicited pMΦs that had been pretreated with piperine for 48 h. Cytokine levels in culture medium were measured using the cytometric bead array together with flow cytometry. All experiments were repeated for three times independently and the data from one representative experiment were presented (mean ± SD, $n=3$). The significance was estimated by Student's *t*-test. ***P* < 0.01.

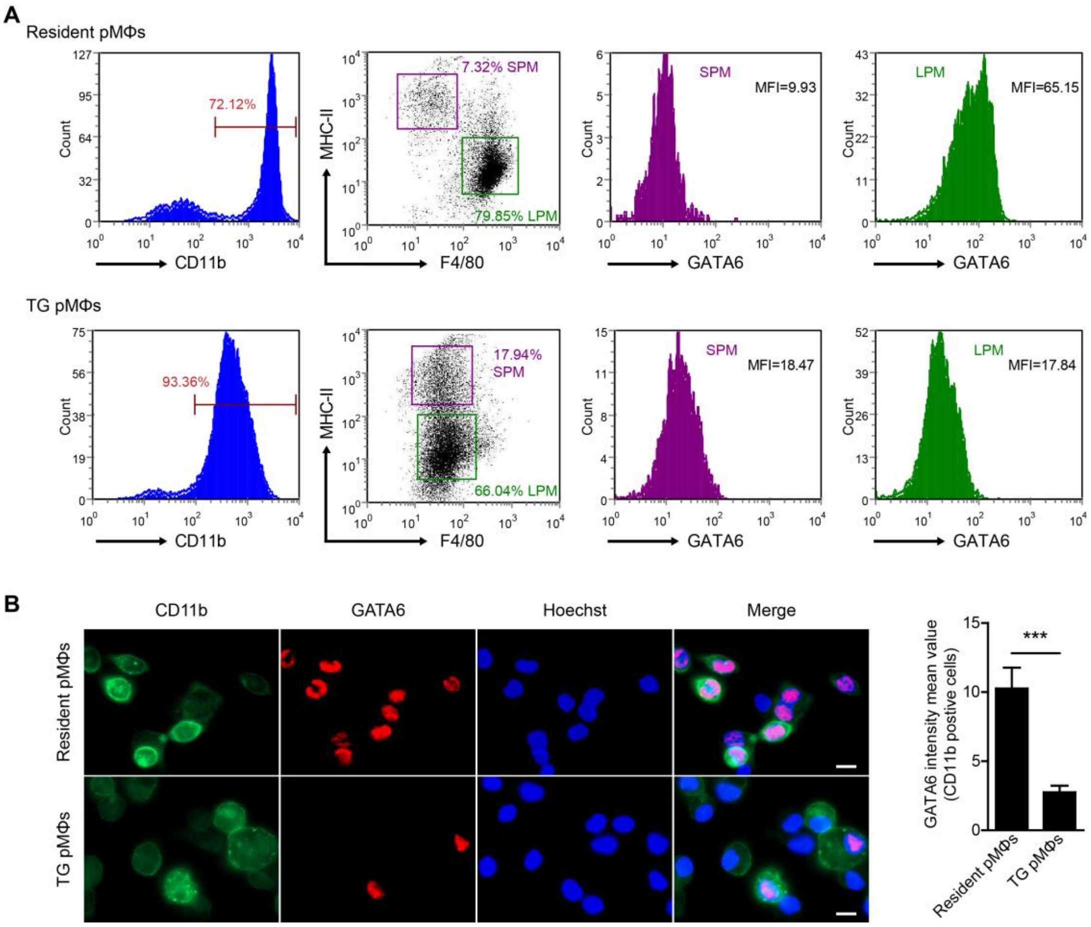

Supplementary Figure S4: Resident and thioglycollate (TG)-elicited pMΦs display differential phenotypes and GATA6 expression. (**A**) Resident and TG-elicited C57BL/6 pMΦs were stained with fluorescent-labeled antibodies (CD11b-FITC, F4/80-PerCP and MHCII-APC). Red blood cells (if there were) were lysed by red blood cell lysis buffer. Cells were fixed in 4% paraformaldehyde and then permeabilized, followed by intracellular GATA6 staining according to the instruction of the manufacturer (Cell Signaling Technology), and flow cytometry analysis. All experiments were repeated for three times independently and one representative set of plots were presented. (**B**) Immunofluorescence microscopy showing the expression levels of GATA6 (red) in $CD11b^{+}$ (green) resident or TG-elicited pMΦs. The nuclei (blue) were revealed by Hoechst33342 staining. Scale bars, 10 μm. In the right panel of (**B)**, data are presented as mean ± SD (*n=10* cells). The significance was estimated by Student's *t*-test. ****P* < 0.001.
